# Treadmill Training Plus Semi-Immersive Virtual Reality in Parkinson’s Disease: Results from a Pilot Study

**DOI:** 10.3390/brainsci13091312

**Published:** 2023-09-12

**Authors:** Massimo Pullia, Laura Ciatto, Giuseppe Andronaco, Concetta Donato, Rosario Ermes Aliotta, Angelo Quartarone, Maria Cristina De Cola, Mirjam Bonanno, Rocco Salvatore Calabrò, Roberta Cellini

**Affiliations:** IRCCS Centro Neurolesi “Bonino-Pulejo”, S.S. 113, Cda Casazza, 98124 Messina, Italy; massimo.pullia@irccsme.it (M.P.); laura.ciatto@irccsme.it (L.C.); giuseppe.andronaco@irccsme.it (G.A.); concetta.donato@irccsme.it (C.D.); rosarioermes.aliotta@irccsme.it (R.E.A.); angelo.quartarone@irccsme.it (A.Q.); mariacristina.decola@irccsme.it (M.C.D.C.); roberta.cellini@irccsme.it (R.C.)

**Keywords:** Parkinson’s disease, neurorehabilitation, semi-immersive virtual reality, C-Mill

## Abstract

Parkinson’s disease (PD) is one of the most common neurodegenerative disorders that causes postural instability and gait alterations, such as reduced walking speed, shorter step length, and gait asymmetry, exposing patients to a higher risk of falling. Recently, virtual reality (VR) was added to a treadmill, in order to promote motor functional recovery and neuroplastic processes. Twenty PD patients were enrolled and randomly assigned to two groups: the experimental group (EG) and the control group (CG). In particular, patients in the EG were trained with the C-Mill, an innovative type of treadmill, which is equipped with semi-immersive VR, whereas the CG performed conventional physiotherapy. Patients in both groups were evaluated through a specific motor assessment battery at baseline (T0) and after the training (T1). Comparing pre-(T0) and post-(T1) treatment scores, in the EG, we found statistical significances in the following outcome measures: 6 Minutes Walking Test (6MWT) (*p* < 0.0005), Timed up and go (TUG right) (*p* < 0.03), Berg Balance Scale (BBS) (*p* < 0.006), Tinetti Scale (TS) (*p* < 0.002), Falls Efficacy Scale- International (FES-I), (*p* < 0.03) Unified PD Rating Scale-III (UPDRS) (*p* < 0.002), and Functional Independence Measure (FIM) (*p* < 0.004). Also, the CG showed statistical significances after the training. Between-group (EG and CG) analysis showed significative statistical differences in 6MWT (*p* < 0.006), BBS (*p* < 0.006), TS (*p* < 0.008), FES-I (*p* < 0.01), and FIM (*p* < 0.009). From our results it emerges that both groups (EG and CG) achieved better outcome scores after the treatment, suggesting that both physiotherapy interventions were effective. However, the EG training using VR seemed to have induced more improvements, especially in gait and balance skills. Then, C-Mill could be a valid adjunctive treatment in the context of gait and balance disturbances, which are very common in the PD population.

## 1. Introduction

Parkinson’s disease (PD) is one of the most common neurodegenerative disorders. It is associated with motor and non-motor alterations, due to the loss of dopaminergic neurons in the pars compacta of substantia nigra [1]. The most common motor alterations include the well-known “cardinal symptoms” (e.g., resting tremor, muscle stiffness, bradykinesia, imbalances, and reduced postural control), in addition to specific gait alterations (such as reduced walking speed, shorter step length, gait asymmetry, and reduced arm swing) [2,3]. These symptoms are the result of the dysfunction of the basal ganglia pathways as well as the hypoactivity of the supplementary motor area (SMA), which is involved in performing motor sequences activated by internal sensory stimuli [4]. Moreover, SMA has a role in the generation of feedforward postural reactions and in executive functions. The hypoactivity of SMA could also explain the postural instability and the dysexecutive syndrome of such patients [5]. To overcome hypoactivity, there is an augmented activity in the lateral premotor cortex, which produces movements in relationship to external stimuli (such as audio-visual biofeedback) [6]. These concepts represent the neuroscientific basis of the usefulness of motor rehabilitation in patients affected by PD. Traditionally, motor rehabilitation is mostly focused on gait training using treadmills, balance and postural exercises, and muscle stretching to reduce body rigidity and prevent joint limitations [7]. In this vein, PD physiotherapy aims to maximize the patient’s functional abilities through the learning of motor strategies and the maintenance of psychophysical conditions. Several studies have shown that motor exercises in addition to audio-visual stimuli, influence positively body posture, balance reactions, and gait [8,9]. In fact, the use of audio-visual stimuli, known as “external cues”, allows an augmented cortical activation of the lateral premotor cortex, which enhances the motor voluntary responses [9]. With the recent advances in technology, rehabilitation has benefitted from a growing use of virtual reality (VR). This consists of a virtual environment in which the user/patient can interact with computer-generated graphics through different degrees of virtual immersion (i.e., non-immersive, semi-immersive, and immersive) [10,11]. Notably, VR provides playful settings that increase the patient’s compliance, amplifying the effect of the rehabilitation itself, and boosting neuroplastic processes through increased repetitions, longer therapy session, and increased motivation [12]. In addition, VR incorporates different kind of feedback, including audio-visual and tactile stimuli that leads to improvements in motor learning through problem solving, besides the high repetitions of movements. In this context, some authors [13,14] investigated the effects of treadmill training combined with VR in PD patients. They suggested that this approach could be a valid way to reduce the risk of falling, also in patients with freezing of gait. According to Gulcan et al. [15], treadmill training plus augmented VR decreased the severity of motor symptoms, improving confidence in balance perception and gait, after 6 weeks of training. However, there is still not enough evidence about the use of treadmills with VR in improving gait and balance functions. To this aim, the C-Mill (Motek, Houten, The Netherlands) [16], a treadmill equipped with semi-immersive VR, could promote neuroplastic processes through task-oriented and high-intensity gait training. Despite these assumptions, the use of C-Mill in PD patients is not well investigated. In this pilot study, we aimed to investigate the role of C-Mill gait training with semi-immersive VR in improving gait and functional motor skills in PD patients.

## 2. Materials and Methods

### 2.1. Study Setting and Participants

A total of 20 patients (7 females and 13 males) with a mean age of 65 ± 8.28 (see Table 1 for more details) attending the IRCCS Neurolesi Center “Bonino-Pulejo”, in Messina (Italy), between October 2021 and December 2022, were enrolled in this pilot study. Inclusion criteria were: (1) diagnosis of PD according to the Movement Disorder Society Clinical Diagnostic Criteria for Parkinson’s Disease; (2) age 50 to 70 years; (3) patients with moderate to advanced disease (2 ≤ Hoehn and Yahr classification grade ≤ 4); and (4) individuals able to walk independently (FAC > 2). PD patients were excluded if they had (1) cognitive, visual, or auditory deficits that impair the comprehension and/or execution of the proposed exercises; (2) presence of comorbidities that prevent upright posture and walking (hypotension); and (3) refusal or impossibility to provide informed consent. Indeed, we excluded for the technological instrumentation issues (related to the C-Mill), PD patients with (1) weight > 135 kg and (2) Height > 200 cm; (3) open lesions or bandages whether in contact with the harness. All experiments were conducted according to the ethical policies and procedures approved by the local ethics committee (IRCCS-ME 37/21). All participants gave their written informed consent.

### 2.2. Procedures

In this pilot study, we enrolled twenty patients with a diagnosis of PD who were able to ambulate and had HY stage 2–4. All patients were randomly assigned to two groups (EG = experimental group and CG = control group), using a web-based app (www.randomization.com) (accessed on 1 January 2023) for block randomization (block size = 4). In detail, ten PD patients underwent the experimental program using the C-Mill (EG—Experimental Group) whereas the other ten received conventional rehabilitation treatments (CG—Control Group). For both groups, the training consisted of 20 sessions lasting 45 min each, 4 times a week for 5 weeks. In detail, training sessions using C-Mill were performed in a dedicated space, called “Innovation Neurorehab Laboratory” in order to standardize each session, through specific settings and protocols. Conventional training sessions were performed indeed in the traditional rehabilitation gym. Additionally, both groups were evaluated by two skilled physiotherapists (M.P. and L.C.) at the beginning (T0) and at the end (T1) of training sessions, through the specific motor battery. The raters were blinded to the patient’s treatment allocation.

#### 2.2.1. Conventional Gait Training (Control Group)

The CG performed a conventional motor rehabilitation program. In detail, specialised physiotherapists (G.A., R.E.A., and C.D.) administered weight-shifting exercises, monopodalic and bipodal balance exercises, and gait training using obstacles, tandem, and slalom walking with different kinds of audio-visual cues. During all sessions, PD patients were manually guided and supervised by the physiotherapists to prevent falls (see Table 2).

#### 2.2.2. The C-Mill Gait Training (Experimental Group)

**Figure 1:**
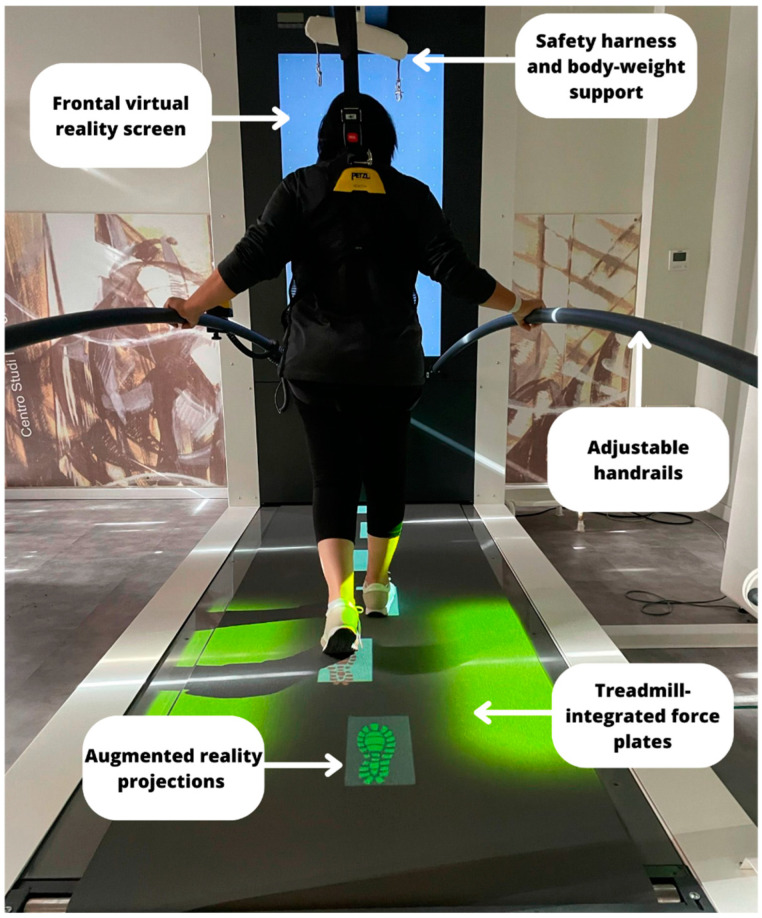
Description of C-Mill components.

The C-Mill system (Motek, Netherlands) [16] consists of an innovative treadmill with a semi-immersive VR system. This device is equipped with a single belt platform with body weight sensors, a harness with a safety rope, a handrail, and a projector, with integrated audio-visual stimuli. During the training session, the PD patient is standing on the treadmill and in front of the big vertical VR screen (see Figure 1).

The visual stimuli can be projected both on the floor and on the VR screen, and they can be set through the CueFors software, which allows physiotherapists to control the various components of the device and monitor the patient’s progress. In addition, the physiotherapist can manually control the device, modifying the visual feedback and acting on specific parameters of gait (e.g., step length, stride length, walking speed, and gait symmetry).

The exercises proposed for the EG included the tandem path, slalom, path with obstacles, and the walk at different speeds. All these gait exercises were accompanied by semi-immersive VR scenarios on the floor, such as the “Arkanoid” (in which the subject has to bounce a ball to destroy a set of bricks, using a virtual paddle), and “Catch” or “Soccer”, in which the subject has to catch different objects or a football while modulating the lower limbs load displacements.

The exercises with the semi-immersive VR frontal screen included “Traffic jam”, which allows the achievement and maintenance of the monopodalic load, and “Italian Alps”, with which the patient can perform walking training including continuous changes of direction by moving to the right and left of the treadmill.

### 2.3. Outcome Measures

Two skilled, blinded to the treatment, physiotherapists (M.P. and L.C.) assessed the PD patients at T0 and T1, using the following specific tests/scales: (i) Berg balance scale (BBS) [17] to assess static and dynamic balance using 14 tasks. The score ranges from 0 to 56, and scores below 40 indicate a moderate-high risk of falling; (ii) Tinetti scale (TS) [18] consists of 16 items, 7 related to gait and 9 related to balance. In particular, a total score of 19 or less indicates a high risk for falling and a score between 19 and 24 indicates a moderate risk; (iii) Falls Efficacy Scale- International (FES-I) [19] evaluates the level of concern relating to falls during 16 different conditions during daily life, and a score between 14 and 28 is associated with a high perception of fear of falling; (iv) Timed up and go (TUG) [20], which is used to determine the fall risk, measuring the patient’s balance, sit to stand ability and walking; (v) ten-meters walking test (10MWT) [21] that is used to evaluate the acceleration during walking; (vi) 6-min walking test (6MWT) [22] to assess patients’ endurance; (vii) Functional independence measure (FIM) [23] was used to evaluate autonomy in daily life activity; and (viii) The Movement Disorder Society-Unified Parkinson’s Disease Rating Scale section III (MDS-UPDRS-III) [24] evaluated the general motor functions (e.g., bradykinesia, postural instability, resting tremor…).

### 2.4. Statistical Analysis

Statistical analyses were performed on the free-source software R 4.1.3 (Vienna, Austria) [25] for Windows and interpreted at the two-tailed significance level of 0.05. Descriptive statistics are reported as mean ± standard deviation (i.e., age, H&Y) or first-third quartiles (i.e., psychometric and outcome measures) for continuous variables, whereas categorial data (e.g., gender) are expressed as frequencies and percentage. Prior to the analysis, all variables were examined for normality, through the Shapiro–Wilk test. Given the non-normal distribution of data (outcome measures and demographic/clinical data) and the small sample size, we performed a non-parametric analysis. At the baseline, the Chi-square test was used to assess gender differences between groups. Differences for age and education, years of illness, H&Y, and clinical outcomes at baseline (T0) were tested through the non-parametric Mann–Whitney test. In addition, we tested outcome measures scores at pre-(T0) and post-(T1) intervention for each group by using the one-tailed Wilcoxon signed rank test (for intra-group analysis) to measure the magnitude of statistical significance of the variation between T0 and T1. While Mann–Whitney’s test was used for the analysis between the experimental and control groups (between-group analysis). In addition, we calculated effect sizes (ES), using Cohen’s test preferable for non-parametric analysis and a small sample size [26].

## 3. Results

All patients completed the training without reporting any side effects, including cybersickness. Demographic, clinical, and outcome measure scores at the baseline (T0) did not show any statistical difference between the two groups (Table 1).

Comparing pre- and post-treatment scores, in the EG, we found statistical significances in the following outcome measures: 6MWT (*p* < 0.0005, ES = 0.93), TUG right (*p* < 0.03, ES = 0.33), BBS (*p* < 0.006, ES = 1.16), TS (*p* < 0.002, ES = 0.89), FES-I (*p* < 0.03, ES = 0.46) UPDRS-III (*p* < 0.002, ES = 0.48), and FIM (*p* < 0.004, ES = 1.13). In addition, the CG showed statistical significances in the same outcome measures: 6MWT (*p* < 0.01, ES = 0.41), BBS (*p* < 0.004, ES = 0.50), TS (*p* < 0.01, ES = 0.59), UPDRS-III (*p* < 0.01, ES = 0.60), and FIM (*p* < 0.005, ES = 0.38) (see Table 3).

**Figure 2:**
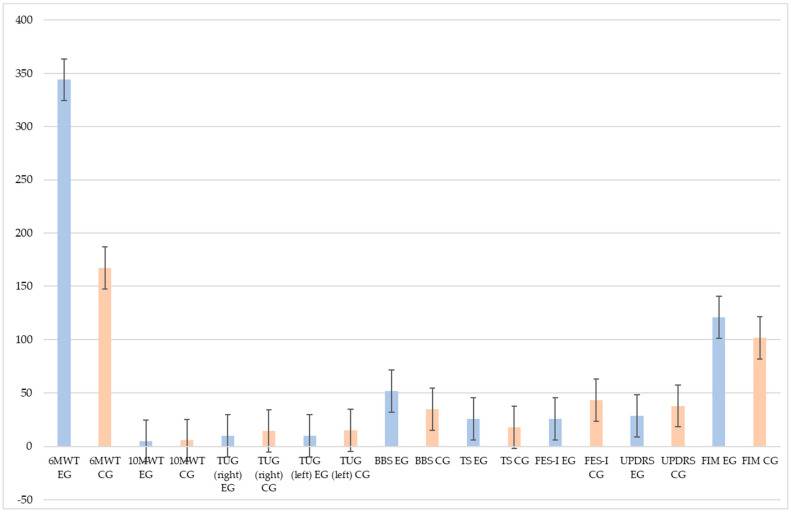
Histogram shows means and standard deviations of the performed outcome measures at post-treatment (T1), comparing the two groups (EG in light blue and CG in orange). Legend: EG (Experimental group), CG (Control group), 6MWT (6-Minutes Walking Test), 10MWT (10-Metre Walking Test), TUG (Timed up and go), BBS (Berg Balance Scale), TS (Tinetti Scale), FES-I (Falls Efficacy Scale-International), UPDRS (Unified Parkinson’s disease rating scale), and FIM (Functional Independence measure). ES (Effect Size) corresponds to between-groups analysis.

The between-group (EG and CG) analysis at post-treatment showed strong significant statistical differences in 6MWT (*p* < 0.006), BBS (*p* < 0.006), TS (*p* < 0.008), FES-I (*p* < 0.01), and FIM (*p* < 0.009) (see Figure 2).

## 4. Discussion

In this pilot study, we investigated the role of C-Mill in improving motor functional outcomes and gait function in a sample of PD patients. From our results, it emerges that both groups (EG and CG) achieved better outcome scores after the training, suggesting that both interventions were effective. In line with our findings, Bekkers et al. [27] found that PD patients with freezing of gait improved their motor functions and postural control using both conventional gait training and treadmill with augmented virtual reality. Since walking is a complex function that requires the integration of both motor and cognitive functions [28], dual-task exercises and/or the use of external cues in both CG and EG could explain the present results. In fact, combined motor and cognitive rehabilitation approaches should be strengthened in clinical practice, at least for ameliorating focused attention, visual processing, and planning during walking [29]. It is noteworthy that the EG, which was trained with semi-immersive VR, achieved better results than the CG, regarding gait and balance skills (6MWT, TS, BBS), risk of falling (FES-I), and global motor functions (FIM). In fact, the use of VR might have the potential to train both motor and cognitive domains (especially executive-attentive and visuospatial functions), and in a safer way than conventional therapy does [30]. Indeed, during conventional training, physiotherapists had to put in more effort because patients tended to fall while avoiding obstacles. Low confidence in balance can limit the performance of daily living activities and exercises during rehabilitation sessions, increasing patients’ concerns about falling [31]. To this aim, the C-Mill may ensure balanced confidence perception through its safety equipment and simple instructions to perform VR exercises. Indeed, the balance exercises through VR incorporate game-like elements and interactive features that promote enjoyment and motivation to participate in training sessions [32,33]. Consequently, a stronger motivation allows the patient to exercise more regularly, precisely, and intensely, and to enhance indirectly motor planning, learning and execution through voluntary drive [34].

During a VR rehabilitation session, the user physically interacts with the virtual environment through an avatar or graphical representation on the screen that mimics the user’s movement [35]. Thus, motor-cognitive interaction can enhance neuroplasticity and motor learning to a greater degree than simple repetitive motor task learning [36]. In this way, the VR-based visual perception of the body induces changes in neural connections that promote the integration between the mirror neuron system and the sensorimotor cortex [37]. The importance of mirror neurons lies in the possibility to predict both the goal of the action and the possible sequence of steps to reach that goal. These assumptions become relevant in the context of motor rehabilitation in PD patients, since they lack motor planning and executive functions, due to the SMA hypofunction [38]. As demonstrated for stroke patients [39], VR feedback during gait training can elicit stronger activation in the frontal-parieto-occipital areas, which are involved in motor intention and planning. These activations seem to be related to improvements in walking ability.

Furthermore, we noticed improvements also in postural control and balance functions (BBS), although the training was most focused on walking, especially in the EG. According to McCrum et al. [40], these improvements in balance through gait training can be related to the hypothesis of the “reverse transfer”. This idea proposes that high-intensity and repeated practice in walking training could enhance non-walking tasks, such as static, balance, and postural stability. Indeed, postural control represents the fundamental basis to produce coordinated locomotor pattern, providing lower limb progression and stability during walking [41]. Some gait exercises, like tandem or heel-to-toe walking, require greater medio-lateral control of the center of mass since the reduced base of support may have a role in improving such balance functions [28].

Moreover, our results showed some improvements also in risk and fear of falling (FES-I). As confirmed by other studies [13,14,15,42], treadmill training with VR can be considered a valid approach to reducing the risk of falls in PD patients. In fact, the high incidence of falls in these patients is primarily induced by cholinergic hypofunction that increases the risk of falls [43]. This idea proposes that gait function is impaired due to the loss of cortical cholinergic inputs, which have a role in the attentional processing of movements. Then, the role of VR in stimulating neuroplasticity in both motor and cognitive pathways may have contributed to reducing the fear of falling, increasing some cognitive aspects of gait function.

This study has some limitations that could limit the generalization of our findings. Firstly, our sample cannot be considered representative of the PD population, due to the small size. In addition, we did not perform a power analysis at priori. However, the study was conceived as a pilot study and the number of subjects enrolled is in line with the study design. Secondly, objective gait analysis was absent. This latter point could be further investigated in future studies with larger samples, and using, for example, motion capture systems to confirm our promising findings more objectively.

Larger RCT could also allow a more robust statistical analysis using parametric tests such as the two-way ANOVA, in the condition of a normal distribution. Finally, future studies could include both motor and cognitive outcomes to understand better the role of C-Mill in improving cognitive aspects of gait, that are fundamental in reducing the risk of falling in such patients.

## 5. Conclusions

In conclusion, this pilot suggests that both conventional and innovative gait training using C-Mill are useful approaches to improve motor functions in PD patients. However, our findings suggest that the use of treadmill plus VR may induce better outcomes, especially concerning endurance and postural control. This is why the C-Mill could be a valid adjunctive treatment in the context of gait and balance disturbances, which are very common in the PD population. Future larger-sample studies are needed to implement the use of C-Mill in clinical practice, both for assessment and treatment issues to guarantee the most effective and tailored rehabilitation approach.

## Figures and Tables

**Table 1:** Demographic and clinical variables of PD sample, at the beginning of the study.

	All Participants (N = 20)	Experimental Group (N = 10)	Control Group (N = 10)	*p*-Value
Age	65 ± 8.28	64.5 ± 10.84	65. 5 ± 10.36	0.75
Gender				0.06
Female	7 (35%)	1 (10%)	6 (60%)	
Male	13 (65%)	9 (90%)	4 (40%)	
Hoehn & Yahr	3 ± 8	2.5 ± 0.78	3.5 ± 0.36	0.07
Years of illness	10.8 ± 7.75	8.3 ± 4.09	13 ± 8.4	0.16
6MWT	180.8 ± 144.4	265 ± 81.07	149.5 ± 105	0.48
10MWT	6.7 ± 4.6	6.7 ± 4.8	6.7 ± 4.6	0.67
TUG				
Right	14.4 ± 10.6	12.1 ± 3.8	16.6 ± 14.5	0.72
Left	15.9 ± 12.5	14.3 ± 9.23	17.4 ± 15.4	0.67
BBS	35.7 ± 15.41	45.1 ± 15.60	36.3 ± 16.15	0.24
TS	15.9 ± 8.26	18.6 ± 7.9	13.2 ± 8.09	0.14
FES-I	38.2 ± 15.64	31.7 ± 12.64	44.8 ± 16.17	0.11
UPDRS	37.7 ± 14.77	37.6 ± 18.6	37.9 ± 10.56	0.67
FIM	102.9 ± 16.99	111.7 ± 18.12	94.1 ± 19.27	0.14

Continuous variables are expressed as mean ± standard deviation, whereas categorial variables are expressed as frequencies and percentages. Legend: 6MWT (6-Minutes Walking Test), 10MWT (10-Metre Walking Test), TUG (Timed up and go), BBS (Berg Balance Scale), TS (Tinetti Scale), FES-I (Falls Efficacy Scale-International), UPDRS (Unified Parkinson’s disease rating scale), and FIM (Functional Independence measure).

**Table 2:** Description of CG and EG exercise training program administered to PD patients.

	CG Exercise Training	EG Exercise Training
Setting	Conventional training with the manual guidance of the physiotherapist.	Innovative training with C-Mill treadmill equipped with semi-immersive VR
Gait training	Gait with obstacles (e.g., bricks, boxes) with different shapes and colours. Gait in tandem with paths created by physiotherapists, using chairs, boxes, traffic cones, sandbags. Walking at different speeds according to audio stimuli provided by therapist (e.g., music and voice of therapist). Walking with frequent direction changes according to audio (e.g., music and voice of therapist) and visual (e.g., coloured tape on the floor) stimuli.	Gait with virtual obstacles projected on the treadmill, with audio-visual stimuli according to target. Gait in tandem with virtual traffic cones, or virtual country paths projected on the treadmill, in addition to audio-visual stimuli. Walking on treadmill at different speeds based on virtual exercises (e.g., “Italian Alps”), projected on frontal screen. Walking on treadmill with frequent lateral direction changes according by virtual projections on the screen (e.g., Arkanoid).

**Table 3:** Medians, first, and third quartile at pre-(T0) and post-treatment (T1), statistical comparisons at T0 and T1 (intra-group analysis) for both groups and at T1 and T1 and effect size (ES).

	Outcome Measures	Median (First–Third Quartile) at T0	Median (First–Third Quartile) at T1	*p*-Value * (T0–T1)	ES
EG	6MWT	260 (183.7–325)	360 (345–381)	**0.0005**	0.93
	10MWT	5.78 (4.56–71.5)	5.27 (4.06–5.85)	0.11	0.33
	TUG				
	Right	9.8 (9.24–15.93)	8.6 (7.19–12.29)	**0.03**	0.58
	Left	10.41 (9.45–18.17)	9.36 (7.5–11.72)	0.05	0.46
	BBS	43 (40–51.23)	52.5 (50.25–55.25)	**0.006**	1.16
	TS	20.5 (15–24.2)	27 (23.5–28)	**0.002**	0.89
	FES-I	29 (21.7–41.5)	23 (21.5–33)	**0.03**	0.46
	UPDRS-III	32 (20.75–58)	25.5 (14.75–44.25)	**0.002**	0.48
	FIM	110.5 (106.5–118.2)	120.5 (117.5–123.7)	**0.004**	1.13
CG	6MWT	45 (0–179.5)	155 (0–294)	**0.01**	0.41
	10MWT	6.43 (2.61–11.89)	6.19 (2.6–8.8)	0.09	0.20
	TUG				
	Right	13.12 (5.67–25.8)	11.35 (5.46–24.4)	0.05	0.15
	Left	15.09 (6.05–25.8)	12.75 (5.2–22.5)	**0.02**	0.15
	BBS	25 (11.5–41.75)	40 (13.25–47.25)	**0.004**	0.50
	TS	11 (7–22.75)	20.5 (8.75–25.25)	**0.01**	0.59
	FES-I	49.5 (32.2–58.2)	47 (28.5–53.25)	0.09	0.10
	UPDRS-III	37 (28.25–46.25)	29 (22.75–42)	**0.01**	0.60
	FIM	95 (79.7–110.2)	104.5 (82–113)	**0.005**	0.38

Legend: 6MWT (6-Minutes Walking Test), 10MWT (10-Metre Walking Test), TUG (Timed up and go), BBS (Berg Balance Scale), TS (Tinetti Scale), FES-I (Falls Efficacy Scale-International), UPDRS (Unified Parkinson’s disease rating scale), and FIM (Functional Independence measure). ES (Effect Size) corresponds to between-groups analysis. ES: 0.2 = small effect; 0.5 = medium effect; 0.8 = large effect [26]. * Significant *p*-values are in bold.

## Data Availability

Data will be available on request to the corresponding author.
